# Self-assembly of nanomicelles with rationally designed multifunctional building blocks for synergistic chemo-photodynamic therapy

**DOI:** 10.7150/thno.68563

**Published:** 2022-01-31

**Authors:** Guidong Gong, Jiezhou Pan, Yunxiang He, Jiaojiao Shang, Xiaoling Wang, Yaoyao Zhang, Guolin Zhang, Fei Wang, Gang Zhao, Junling Guo

**Affiliations:** 1 BMI Center for Biomass Materials and Nanointerfaces, College of Biomass Science and Engineering, School of Chemistry Engineering, Sichuan University, Chengdu, Sichuan 610065, China.; 2 Center for Natural Products Research, Chengdu Institute of Biology, Chinese Academy of Sciences, Chengdu, Sichuan 610041, China.; 3 Key Laboratory of Birth Defects and Related of Women and Children of Ministry of Education, The Reproductive Medical Center, Department of Obstetrics and Gynecology, West China Second University Hospital, Sichuan University, Chengdu, Sichuan 610041, China.; 4 State Key Laboratory of Polymer Materials Engineering, Sichuan University, Chengdu, Sichuan 610065, China.

**Keywords:** Nanomicelle, coordination, self-assembly, oxygen self-supply, chemo-photodynamic therapy.

## Abstract

**Rationale**: The combination of photosensitizers, oxygen supply agents, and adjuvant therapy drugs in a single nano-drug delivery system for photodynamic therapy (PDT) has been showing great promises to overcome the inherent challenges of PDT for tumor treatment. However, the complicated preparation of integrating multiple components hampers their further developments. Here, we describe a self-assembly nanomicelle with rationally designed building blocks, which shows a high efficiency of synergistic chemo-photodynamic therapy in the animal modal.

**Methods**: The nanomicelle was prepared by a coordination-driven self-assembly based on a rationally designed ferrocene cyclopalladated compound coupled with photosensitizers and hyaluronic acid (referred to as FCP-Tph/HA). The morphology, targeting drug delivery, pharmacokinetics, hemolysis, and multimodal synergistic therapy of FCP-Tph/HA were investigated.

**Results**: The formation of nanomicelles presents a low hemolysis rate and a prolonged blood circulation time. FCP-Tph/HA possesses an enhanced antitumor effect *in vitro* through the specific binding of HA to CD44 and combining chemotherapy with oxygen self-supplying PDT. Simultaneously, the nanomicelle facilitates a significantly improved antitumor efficacy (>90% tumor regression) on a breast cancer model *in vivo*.

**Conclusion**: Our results present a modular self-assembled nanomicellar platform with synergistic chemo-photodynamic therapy for challenging PDT-based tumor treatment.

## Introduction

Photodynamic therapy (PDT) has been shown as a rapidly growing and versatile approach for treating numerous diseases due to the advantages of relatively low systematic toxicity, non-invasion, and high efficacy 1-3. However, nonselective aggregation of photosensitizers and failure in the hypoxic tumor region severely diminish the efficacies of PDT in tumor therapy 4,5. To address this key challenge of PDT in treating tumors (i.e., the limited efficacy of PDT resulted from the hypoxic tumor microenvironment), nano-drug delivery PDT systems (nanoPDTs) have been explored with the capability of oxygen generation in situ or direct oxygen delivery, including by supplying oxygen with the decomposition of endogenous hydrogen peroxide, generating oxygen via photosynthesis, and transporting oxygen with hemoglobin 6-10.

The combination of PDT with other therapies (e.g., chemotherapy, immunotherapy, and photothermal therapy) exhibits an enhanced therapeutic efficacy due to their synergistic effects of multiple biological mechanisms of tumor treatments 7,10-16. Thus, developing an effective nanoPDT with the integration of adjuvant therapies offered a promising approach for tumor treatment 13,17. However, the integration of multiple components with diverse molecular structures (i.e., photosensitizers, oxygen supply agents, and synergistic drugs) in a single nanoPDT leads to the increased complexity of the preparation process and unpredictable side effects 18,19. Therefore, it is highly desirable to develop a next-generation nanoPDT system to achieve a facile preparation and an enhanced antitumor effect 20-22.

**Figure 1:**
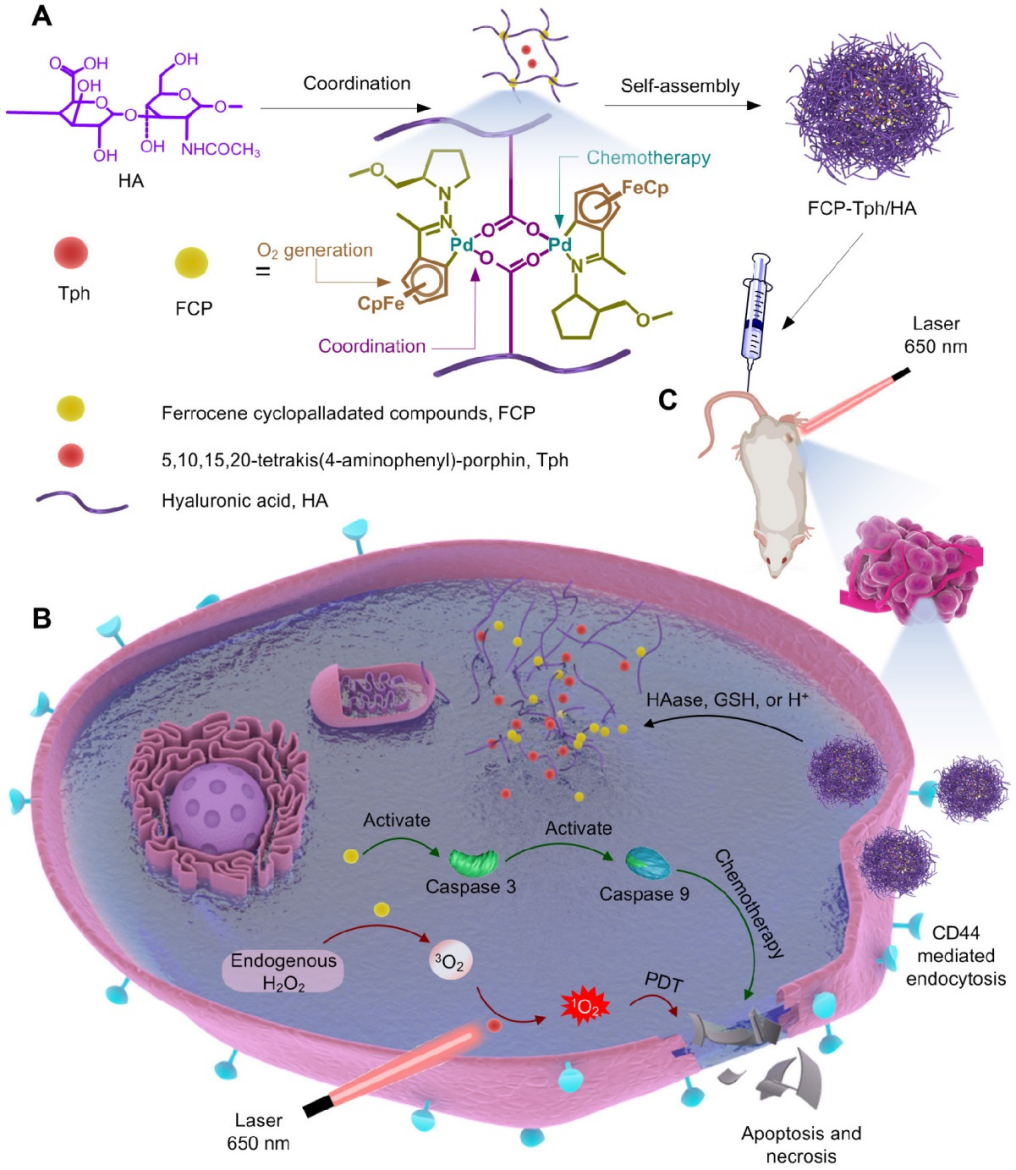
**Schematic representation of FCP-Tph/HA for chemo-photodynamic therapy in tumors.** A) Preparation of FCP-Tph/HA by a coordination-driven self-assembly. B) The molecular mechanism of FCP-Tph/HA for synergistic chemo-photodynamic therapy in tumor treatments. C) Active targeting delivery of FCP-Tph/HA by the specific binding of HA and CD44.

Herein, we report a nanoPDT micelle constructed by a rationally designed ferrocene cyclopalladated compound (FCP) coupled with a photosensitizer 5,10,15,20-tetrakis(4-aminophenyl)-porphin (Tph, Figure S1A) and hyaluronic acid (HA) in an integrative system (referred to as FCP-Tph/HA, Figure 1). The FCP building block possesses a range of functions that can be used as a chemotherapeutic drug, an oxygen supply agent, and a modular assembling building block (Figure 1A) 23-25. Specifically, our studies show that FCP exhibits the capability of inducing apoptosis in cancer cells through a caspase-dependent pathway (Figure 1B). Moreover, due to the switchability of the ligands, the acetic acid coordinated-FCP can form micelles with macromolecules containing carboxyl groups like HA 25. Besides, HA is well-known to specifically bind to overexpressed CD44 on tumor cells 13,24, facilitating an active targeting ability to tumors (Figure 1C) 6,27,28. Our results showed that the coordination between HA and FCP played a key role in the nanomicellar formation mechanism. Cellular uptake experiments and biodistribution experiments confirmed the active tumor-targeting ability of FCP-Tph/HA. Hemolytic tests and pharmacokinetic experiments indicated the formation of nanomicelle significantly reduces the hemolytic rates and prolongs the blood circulation time. Finally, FCP-Tph/HA performed a synergistic chemo-photodynamic therapeutic effect *in vivo*, showing a high efficacy of tumor regression (~ 90%) on a breast cancer model.

## Methods

### Oxygen generation assay

FeCl_3_·6H_2_0 (2.7 mg, 1 mmol), FCP (10.2 mg, 1 mmol), and Fc (1.9 mg, 1 mmol) were added into 3 tubes (15 mL), respectively. Followed by adding 10 mL H_2_O_2_ solution (3% of mass ratio, pH = 7.1) and the O_2_ concentration probes. The O_2_ concentration of the solution in each group was recorded by every 30 s in the first 6 minutes (T = 25°C).

### Detection of singlet oxygen

Singlet oxygen sensor green reagent (SOSG, specifically react with ^1^O_2_, and show strong fluorescence at 530 nm under excitation at 494 nm, [SOSG] = 5 μM) dissolved in methanol was incubated with different samples (H_2_0, 1 μM FCP, 1 μM Tph, and FCP-Tph/HA, [FCP] = 1 μM in FCP-Tph/HA) and H_2_O_2_ ([H_2_O_2_] = 0.25 mol/L) to measure ^1^O_2_ generation after laser irradiation (650 nm, 200 mW/cm^2^, 5 min) under nitrogen atmosphere. The generation of ^1^O_2_ was determined by measuring the SOSG fluorescence signal at 530 nm under 494 nm excitation.

### Preparation of FCP/HA, FCP-C-6/HA, FCP-DIR/HA, and FCP-Tph/HA

10 mg (10 μmol) FCP and 59 mg HA were dissolved in 0.5 mL DMSO and 3.0 mL deionized water, respectively. The FCP solution was added dropwise to HA solution under ultrasonic conditions. The mixed solution was sonicated for another 30 min, followed by stirring at room temperature for 72 h. The nanomicelle FCP/HA was obtained after dialysis for 24 h and stored at 4°C for future use.

The FCP-C-6/HA, FCP-DIR/HA, and FCP-Tph/HA were prepared in an analogical way. Of a certain amount (10 μmol) of Coumarin 6 (C-6), 1,1'-dioctadecyl-3,3,3',3'-tetramethylindotricarbocyanine iodide (DIR) or Tph was mixed with FCP in DMSO. HA was dissolved in deionized water. The FCP with C-6, DIR, or Tph were dropped to the HA solution under dark conditions. The mixed solution was sonicated for another 30 min, followed by stirring at room temperature for 72 h. The nanomicelle FCP/HA was obtained after dialysis for 24 h and stored at 4°C for future use.

### Biological studies

MDA-MB-2321 cells and NIH 3T3 cells were maintained in DMEM (Invitrogen, USA) containing 10% fetal calf serum (Invitrogen) and 1% penicillin/streptomycin at 37°C in a 5% CO_2_ atmosphere. 4T1 cells were placed in Roswell Park Memorial Institute 1640 (RPMI 1640, Invitrogen) containing 10% fetal calf serum (Invitrogen) and 1% penicillin/streptomycin at 37°C in a 5% CO_2_ atmosphere.

### Cell viability assay

Cells were seeded in 96-well plates at a density of 5 × 10^3^ cells/well with 100 μL medium. Cultured cells were treated with FCP, FCP/HA, HA + FCP/HA (Addition of 100 μg/L HA firstly, followed by the FCP/HA after 1 h), or cisplatin at the indicated concentrations. After 72 h, the remaining medium was discarded and another 100 μL fresh medium with 10% cell counting kit-8 (CCK-8, Solarbio) was added to the medium, and the cells were incubated for 2 - 4 h until the color of the control group turned bright yellow. The A450 intensity was measured using a Thermo Scientific Varioskan Flash multimode reader.

Cells were seeded in 96-well plates at a density of 5 × 10^3^ cells/well with 100 μL medium. Cultured cells were treated with FCP-Tph/HA, Tph and FCP-Tph/HA - O_2_, Tph - O_2_ (1% O_2_ atmosphere) for 4 h, and then the cells were washed with PBS for 4 times. Fresh medium was added and irradiated by a laser with 650 nm, 200 mW/cm^2^ for 10 min. After 20 h, the remaining medium was discarded and another 100 μL fresh medium with 10% CCK-8 (Solarbio) was added to the medium, and the cells were incubated for 2 - 4 h until the color of the control group turned bright yellow. The A450 intensity was measured using a Thermo Scientific Varioskan Flash multimode reader.

### Cellular uptake assay (ICP-MS)

MDA-MB-231 and 4T1 cells were used to evaluate the cellular uptake of FCP/HA. Briefly, 5 × 10^5^ MDA-MB-231 and 4T1 cells were pre-seeded on a round coverslip in 6 well plates for 12 h, FCP/HA and HA + FCP/HA (100 μg/L HA was firstly added, and incubated for 1 hour, followed by the addition of FCP-C-6/HA) were added at an equivalent concentration of FCP (5 μM) and incubated 8 h, the cells were harvested and counted. Next, the cells were digested by aqua regia, and the concentrations of Pd were tested by ICP-MS.

### Cellular uptake assay (confocal laser scanning microscope)

MDA-MB-231, 4T1, and NIH 3T3 cells were used to evaluate the cellular uptake of FCP/HA. Briefly, 5 × 10^5^ MDA-MB-231, 4T1, and NIH 3T3 cells were pre-seeded on a round coverslip in 6 well plates. Free C-6, FCP-C-6/HA (concentration of FCP, 5 μM), and HA + FCP-C-6/HA (concentration of FCP, 5 μM) were added at an equivalent concentration of C-6 (200 ng/mL) after 4 h at 37°C. The cells were then washed 3 times with cold DPBS and fixed with 4% formaldehyde. Coverslips were mounted on slides and examined by Leica TCS SP8 confocal laser scanning microscopy (Leica Microsystems Inc., Buffalo Grove, USA).

### ROS level assay

The ROS level in MDA-MB-231 cells after incubated with Tph ([Tph] = 5 μM), Tph upon 650 nm light irradiation with 200 mW/cm^2^ (marked as Tph + PDT, [Tph] = 5 μM), Tph with 650 laser and 1% O_2_ (marked as Tph + PDT - O_2_, [Tph] = 5 μM, other experiments that did not specify the oxygen concentration were all carried out at 21% oxygen concentration), FCP (5 μM), FCP/HA ([FCP] = 5 μM), FCP-Tph/HA ([FCP] = 5 μM, [Tph] = 5 μM), FCP-Tph/HA with 650 nm, 200 mW/cm^2^ laser (marked as FTP-Tph/HA + PDT, [FCP] = 5 μM, [Tph] = 5 μM), and FCP-Tph/HA with 650 nm, 200 mW/cm^2^ laser and 1% O_2_ (marked as FCP-Tph/HA + PDT - O_2_, [FCP] = 5 μM, [Tph] = 5 μM) was assessed with ROS assay kit (Beyotime). In brief, MDA-MB-231 cells cultured in 6-well plate at 4 × 10^5^ cell/well were treated for 4 h with DMSO (as control) and Tph, Tph + PDT (irradiated with a 650 nm, 200 mW/cm^2^ laser for 10 min), Tph + PDT - O_2_ (irradiated with a 650 nm, 200 mW/cm^2^ laser for 10 min), FCP, FCP/HA, FCP-Tph/HA, FCP-Tph/HA + PDT (irradiated with a 650 nm, 200 mW/cm^2^ laser for 10 min) and FCP-Tph/HA + PDT - O_2_ (irradiated with a 650 nm, 200 mW/cm^2^ laser for 10 min) at the concentration of 5 μM FCP and 5 μM Tph. After that, the cells were washed with PBS three times and 1 mL diluted DCFH-DA. After 20 min, the cells were washed with PBS three times and fluorescence intensity (Em = 525 nm, Ex = 488 nm) was measured by the Thermo Scientific Varioskan Flash multimode reader.

MDA-MB-231 cells cultured in a 6-well plate at 4 × 10^5^ cell/well were treated for 2 h with DMSO (as control, two groups) and FCP-Tph/HA ([FCP] = 5 μM, [Tph] = 5 μM) under the hypoxia atmosphere. After that, the cells were washed with PBS three times and 1 mL of RPMI 1640 with 10 mM H_2_O_2_ was added and incubated for another 20 min. Next, the cells were washed with PBS three times to remove the rest of H_2_O_2_ and 1 mL diluted DCFH-DA was added. After 20 min, the cells were washed with PBS three times and the fluorescence intensity (Em = 525 nm, Ex = 488 nm) was measured by Thermo Scientific Varioskan Flash multimode reader (Thermo Fisher Scientific, USA).

### Caspase 3 and 9 activity assays

The activities of Caspase 3 and 9 in MDA-MB-231 cells were assessed based on the specific protease-peptide substrate chromogenic reaction. In brief, MDA-MB-231 cells cultured in 6-well plates at 4 × 10^5^ cell/well were treated for 12 h with PBS (as control) and FCP (5 μM) or FCP/HA ([FCP] = 5 μM). After that, the cells were harvested, lysed, and centrifugated. Then, aliquots of supernatants were collected and incubated with the peptide substrates of Caspase 3 (Ac-DEVD-pNA) and 9 (Ac-LEHD-pNA) (Enzo Life Sciences, Inc., USA), respectively. The activities of Caspase 3 and 9 were determined based on the absorbance at 405 nm by Thermo Scientific Varioskan Flash multimode reader. The total protein content of each sample was determined to normalize the obtained values by a BCA protein assay kit (Bestbio, China), and the activity ratio was calculated as compared to the blank control.

### Flow cytometry assay

A total of 2 × 10^5^ MDA-MB-231 cells were plated in a 6-well plate for 24 h and then treated with DMSO, FCP ([FCP] = 5 μM for 4 h, washed with PBS for 4 times and incubated for another 20 h), FCP/HA ([FCP] = 5 μM, 4 h, washed with PBS for 4 times and incubated for another 20 h), Tph ([Tph] = 5 μM, 4 h, washed with PBS for 4 times and incubated for another 20 h), Tph + PDT ([Tph] = 5 μM for 4 h, and washed with PBS for 4 times, irradiated with a 650 nm, 200 mW/cm^2^ laser for 10 min and incubated for another 20 h), Tph + PDT - O_2_ ([Tph] = 5 μM for 4 h, and washed with PBS for 4 times, irradiated with a 650 nm laser with 200 mW/cm^2^ for 10 min and incubated for another 20 h, all the operation and incubation were in 1% O_2_ atmosphere), FCP-Tph/HA ([FCP] = 5 μM, [Tph] = 5 μM for 4 h, and washed with PBS for 4 times and incubated for another 20 h), FCP-Tph/HA + PDT ([FCP] = 5 μM, [Tph] = 5 μM for 4 h, and washed with PBS for 4 times, irradiated with a 650 laser with 200 mW/cm^2^ for 10 min and incubated for another 20 h), FCP-Tph/HA + PDT - O_2_ ([FCP] = 5 μM, [Tph] = 5 μM for 4 h, and washed with PBS for 4 times, irradiated with a 650 laser with 200 mW/cm^2^ for 10 min and incubated for another 20 h, all the operation and incubation were in 1% O_2_ atmosphere). Next, all the treatment groups were washed with PBS 4 times and changed to a fresh medium. After incubation, the cells were harvested and washed with ice-cold PBS. The apoptosis ratio was performed with an annexin V-FITC Apoptosis Detection Kit (Beyotime).

### Hemolysis assay

The hemolysis rate experiment was carried out to evaluate the erythrocyte compatibility of FCP, FCP/HA, and FCP-Tph/HA. The fresh rat blood was collected in vacuum tubes containing citric acid as an anticoagulant. Then, the blood (8.0 mL) was diluted by PBS (0.01 M, 10.0 mL). Samples were immersed in PBS (10.0 mL) as the experimental groups. The groups of negative control and positive control were treated with 10.0 mL of PBS and deionized water, respectively. All the groups were incubated at 37°C for 30 min. Then, the diluted blood (200 μL) was added to each group and incubated at 37°C for 4 h. Finally, the supernatant was detected at 545 nm by a UV-Vis spectrophotometer.

### Pharmacokinetics

Sprague-Dawley rats (200 - 250 g) were purchased from Dashuo Laboratory Animal Technology, Ltd. (Chengdu, China) and were kept on a 12 hours day/night cycle with free access to food and water. All the experiments and procedures were performed in accordance with the National Institutes of Health guide for the care and use of Laboratory Animals (NIH Publications No. 8023, revised 1978). All animal experiments were approved by the Experimental Animal Management and Ethics Committee of West China Second University Hospital (Approval number: 2020-029). All rats were randomly assigned to two treatment groups (6 rats/group). FCP and FCP/HA were administrated by intravenous (I.V.) injection with an equivalent FCP dose of 10 mg/kg. At timed intervals, 0.3 mL of blood was collected and centrifuged. The concentration of FCP in plasma was analyzed by Waters UPLC-Xevo™ UPLC-TQ MS (Waters Co., Ltd.).

### *Ex-vivo* biodistribution assay

4T1 tumor-bearing Balb/c mice (n = 3) were utilized to investigate the biodistribution of FCP/HA. When the tumor volume reached approximately 500 mm^3^, free DiR, DiR labeled FCP/HA (ie., FCP-DIR/HA) were administrated via the tail vein at a dose equivalent to 1 mg/kg of DiR. The mice were killed after 24 h post-injection and the major organs (heart, liver, spleen, lung, and kidney) and tumors were harvested and imaged using an IVIS spectrum small-animal imaging system (IVIS Lumina Series III, PerkinElmer).

4T1 tumor-bearing Balb/c mice (n = 3) were utilized to investigate the biodistribution of FCP-DIR/HA. When the tumor volume reached approximately 500 mm^3^, FCP-DIR/HA was administrated via the tail vein at a dose equivalent to 1 mg/kg of DiR. The mice were killed after 0, 6, 12, 24, and 48 h post-injection, and the tumors were harvested and imaged using an IVIS spectrum small-animal imaging system (IVIS Lumina Series III, PerkinElmer).

### Mouse xenograft model

Female Balb/c mice (aged five weeks) were purchased from Dashuo Laboratory Animal Technology, Ltd. (Chengdu, China) and were kept on a 12 h day/night cycle with free access to food and water. All the experiments and procedures were performed in accordance with the National Institutes of Health guide for the care and use of Laboratory Animals (NIH Publications No. 8023, revised 1978). All animal experiments were approved by the Experimental Animal Management and Ethics Committee of West China Second University Hospital (Approval number: 2020-029). 4T1 cells (5 × 10^6^ cells/mouse) were injected into the right-back of mice in serum-free RPMI 1640 (100 μL). The tumor volume was calculated using the formula: 
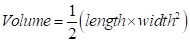
. When the tumor volume was approximately 100 mm^3^, mice were randomly segregated into six groups (five mice per group) and treated with normal saline, HA, Tph, FCP, FCP/HA, and FCP-Tph/HA, respectively. The tumor size was measured every third day with a caliper. After 15 days, mice were sacrificed. The tumors and major organs were collected. The formalin-embedded tissues were stained by H&E to identify the pathological change of major organs.

### Statistical analysis

Statistical analyses were performed with GraphPad Prism 5.0 software (GraphPad, La Jolla, CA, USA). All experiments were repeated at least thrice, and representative results are presented. The data were compared by one-way ANOVA followed by Dunnett's post-hoc test. The differences were considered statistically significant when p < 0.05.

## Results and Discussion

**Figure 2:**
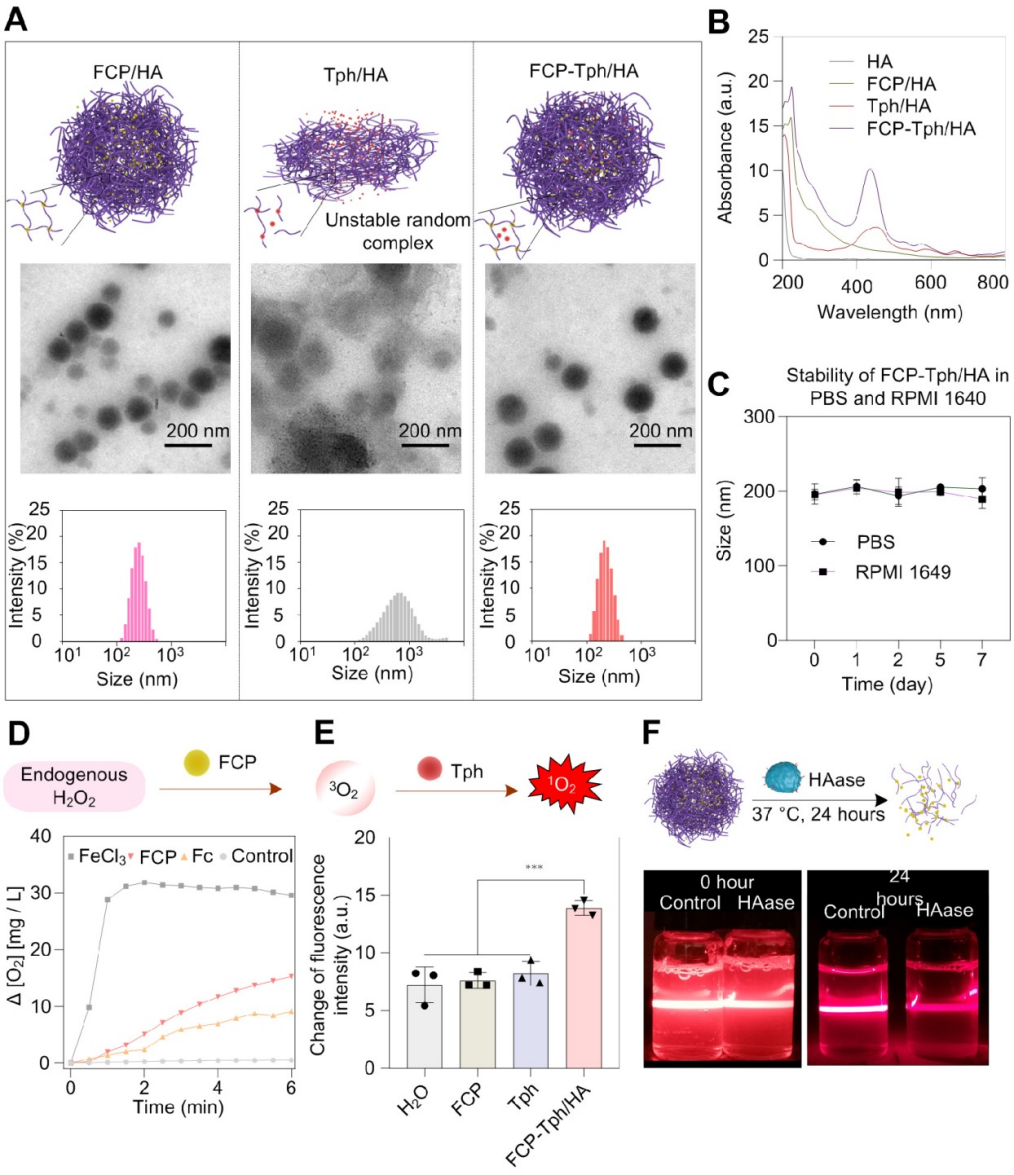
** Characterization of micelles.** A) TEM images and corresponding DLS analysis of FCP/HA, Tph/HA, and FCP-Tph/HA. Scale bar, 200 nm. B) UV-Vis spectra of HA, FCP/HA, Tph/HA, and FCP-Tph/HA. C) Size of FCP-Tph/HA in PBS and RPMI 1640 at different time. D) The plot of oxygen concentration versus time when FeCl_3_, Fc, FCP were treated with hydrogen peroxide solution (3% of the mass fraction). E) Change in fluorescence of SOSG after being treated with H_2_O, FCP, Tph, and FCP-Tph/HA with H_2_O_2_ (1% of the mass fraction). F) Tyndall effect of the nanomicelle FCP/HA stimulated by HAase for 24 h at 37°C.

FCP was obtained through a two-step synthesis. Briefly, the ligand acetylferrocene hydrazone was synthesized by a condensation reaction between acetylferrocene and 1-amino-2-(methoxymethyl)pyrrolidine in dry benzene. Acetylferrocene hydrazone was mixed with Pd(OAc)_2_ at a 1:1 molar ratio in dry methanol for 24 h and afforded FCP at a moderate yield (Figure S1B). The preparation process of micellar carriers was being explored by coordinating HA and FCP at different masses (Table S1). Three micelles prepared from 10 kD HA with less than 220 nm size were ideal carriers for the construction of nanoPDT 29,30. Ultimately, the micelle (marked as FCP/HA) at a 5.9:1 feed mass ratio of HA and FCP was selected as the carrier for Tph because of the low polymer dispersion index (PDI, 0.053), low zeta potential (-34.7 mV), sufficient embedding rate (ER, 12.82%) and high drug loading (DL, 86.78%) (Table S2). When preparing the nanomicelle with the cargo Tph (i.e., FCP-Tph/HA), the size, PDI, zeta potential, and DL of Tph were 224.9 nm, 0.127, -28.6 mV, and 84.08% respectively (Figure 2A). We further simplified the construction of nanoPDT by mixing Tph with HA directly (referred as to Tph/HA). The dynamic light scattering (DLS) result showed a large size of 546.1 nm and a high PDI of 0.271, which was not qualified as a nanoPDT. The results also confirmed the essential role of FCP in the construction of nanoPDT. The presence of a characteristic band at around 435 nm indicated the successful entrapment of Tph in the FCP-Tph/HA (Figure 2B). In addition, FCP-Tph/HA could keep stable in PBS and cell culture medium RPMI 1640 for 7 days, which indicates the high potential of FCP-Tph/HA as a high-quality nano-drug (Figure 2E). TEM images of FCP/HA and FCP-Tph/HA showed nanoscale spherical micelles, while Tph/HA showed random complexes, which confirmed the DLS results that the addition of FCP was necessary for the construction of nanoPDT (Figure 2A).

Hydrogen peroxide with a high level in the solid tumor microenvironment offers a chance to self-supply oxygen for PDT 7. To evaluate the oxygen supply capability of FCP, the oxygen conversion test of hydrogen peroxide was tested using FeCl_3_ and ferrocene (Fc) as controls. The concentration of oxygen increased with the addition of FCP to hydrogen peroxide solution (Figure 2D), which demonstrated the potential of FCP as an oxygen generation agent for PDT in the hypoxic tumor microenvironment 6. We evaluated the ^1^O_2_ generation in the simulated hypoxia microenvironment with H_2_O_2_ using SOSG. The concentration of ^1^O_2_ (represented by the increased fluorescence intensity) in the SOSG solution after being treated with FCP-Tph/HA was significantly high than that of the SOSG solution after being treated with H_2_O_2_, FCP, and Tph (Figure 2E). Considering the effective generation of oxygen via the catalysis of FCP on H_2_O_2_, the production of ^1^O_2_ by FCP-Tph/HA with H_2_O_2_ presented the highest fluorescence change. This observation also indicated that the concurrence of FCP and Tph could possibly promote the PDT efficacy of FCP-Tph/HA *in vitro* and *in vivo*.

We evaluated the decomposition of FCP/HA by adding hyaluronidase (HAase) to mimic the degradation of the nanomicelle *in vivo*. The Tyndall effect and DLS of FCP/HA were tested before and after HAase was added. After being incubated with HAase for 24 h at 37°C, the nanomicelle witnessed a significantly weaker scattering ability of the laser and a wider particle size distribution (Figure 2F, and Figure S4). The results indicated that FCP/HA disintegrated with the addition of HAase, suggesting the satisfactory biodegradability of FCP/HA as a nanoPDT 29-32. In addition, both acid (pH 5.4) and glutathione (GSH, 5 mM) could lead to the decomposition of FCP-Tph/HA. These results indicated that the acidic cytoplasm and the intracellular GSH could also accelerate the release of FCP and Tph after the nanomicelles internalized by the cancer cells (Figure S5).

**Figure 3:**
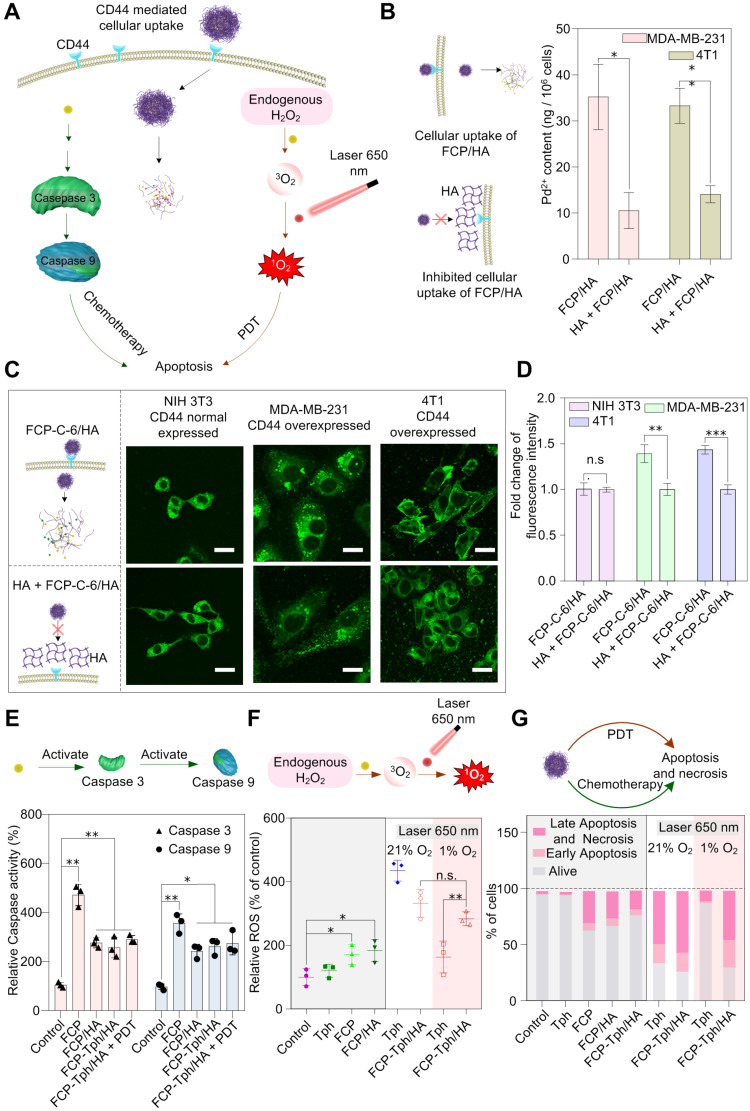
***In vitro* antitumor activity studies.** A) Schematic illustration of the *in vitro* antitumor mechanism studies of FCP-Tph/HA. B) The content of Pd^2+^ in MDA-MB-231 and 4T1 cells tested by ICP-MS. C) Representative images of cellular uptake incubated with FCP-C-6/HA and HA + FCP-C-6/HA for 4 h, scale bar represents 20 μm. D) Fold change of fluorescence intensity account after incubation with FCP-C-6/HA, and HA + FCP-C-6/HA. E) Caspase 3 and Caspase 9 activation in 4T1 cells after treatment with FCP, FCP/HA, FCP-Tph/HA with or without laser irradiation (10 min) for 12 h. F) ROS level in MDA-MB-231 after treatment with different conditions for 6 h. G) Different cell percent obtained from an apoptotic study using annexin-V FITC and propidium iodide (PI) after different treatment conditions. All values are presented as the mean ± SD (n = 3). (*) p < 0.05, (**) p < 0.01, (***) p < 0.001, (n.s.) not significant.

The cytotoxicity of FCP/HA and FCP was assessed on breast cancer cell lines (MDA-MB-231 and 4T1, CD44 positive), and a mouse embryonic fibroblast cell line (NIH 3T3, CD44 negative) with taking Cisplatin as a positive control (Figure 3A and Table S3). The IC_50_ values of FCP for MDA-MB-231 and 4T1 cell lines were significantly lower than that of Cisplatin, which indicated the potential of FCP being used as a chemotherapeutic agent for tumor treatment. Besides, the IC_50_ values of MDA-MB-231 and 4T1 in the HA pretreated group (treated with 100 μg/L HA for 1 hour and then treated with FCP/HA, marked as HA + FCP/HA) were almost 2 - 3 times higher than those in the FCP/HA treatment group. However, no significant difference was observed on the IC_50_ values of NIH 3T3 treated with FCP/HA or HA + FCP/HA. This can be explained that after assembled with HA, the cytotoxicity of FCP was mainly induced by the CD44 mediated cellular uptake in CD44 overexpressed cell lines MDA-MB-231 and 4T1. If the HA receptors CD44 were blocked by over HA, the cellular uptaking of the FCP/HA was reduced and higher IC_50_ values occurred 29. The results also ascribed the micelle containing HA possessed an active targeting drug delivery ability due to the specific binding of HA with the overexpressed CD44 on MDA-MB-231 and 4T1 cells. The content of Pd^2+^ in cells treated with FCP/HA or HA + FCP/HA was measured by ICP-MS. The result illustrated that the Pd^2+^ contents in both MDA-MB-231 and 4T1 cells incubated with HA + FCP/HA were significantly lower than that in cells incubated with FCP/HA, which supported the reduced cellular uptake of FCP/HA micelles due to the inhibition of CD44 by HA pretreatment (Figure 3B). Furthermore, to verify the ICP-MS results, the cellular uptake test with Coumarin 6 labeled nanomicelle (marked as FCP-C-6/HA) was carried out and analyzed by laser scanning confocal microscope 31,32. After incubated with FCP-C-6/HA, or firstly HA for 1 hour and then FCP-C-6/HA (marked as HA + FCP-C-6/HA), the images of NIH 3T3, 4T1, and MAD-MB-231 were collected. As shown in Figure 3C, D, the fluorescence intensities of 4T1 and MDA-MB-231 cells incubated with FCP-C-6/HA were higher than that of cells incubated with HA + FCP-C-6/HA. However, the fluorescence intensities of NIH 3T3 cells incubated with FCP-C-6/HA were not different from that of cells incubated with HA + FCP-C-6/HA, which indicated that the pretreated HA could reduce the cellular uptake of FCP/HA in CD44-positive cells, but not in CD44-negative cells. The apoptosis-related proteins were examined to clarify the cytotoxicity mechanism of FCP/HA micelles. The increase of Caspase 3 and Caspase 9 activities in 4T1 cells after being treated with FCP, FCP/HA, and FCP-Tph/HA with or without laser irradiation indicated that FCP could induce a caspase-dependent apoptosis pathway in 4T1 cells (Figure 3E) 23.

Moreover, to demonstrate the ability of FCP to supply oxygen during the PDT, the cell viability was tested under normoxic and hypoxic conditions on the MDA-MB-231 cell line incubated with FCP-Tph/HA and Tph for 4 h (Figure S6). Both FCP-Tph/HA and Tph showed a similar level of high cytotoxicity to MDA-MB-231 cells under normoxic conditions, while the cytotoxicity of FCP could be neglected. Under hypoxic conditions, ~40% of cells survived after being treated with FCP-Tph/HA (1 μM FCP and 1 μM Tph), while ~80% of cells survived when treated with Tph (1 μM). The results suggested that oxygen was indispensable in PDT, and FCP-Tph/HA could overcome the issue of PDT failure in a hypoxic environment 7. The ROS level was measured to clarify the synergy advantages of the oxygen self-supplied nanoPDT FCP-Tph/HA for tumor therapy 23,33. As shown in Figure 3F, in the absence of 650 nm laser irradiation under the normoxic atmosphere, Tph could not improve the ROS level in MDA-MB-231 cells. However, with laser irradiation, the ROS level dramatically increased. The increase was only observed under the normoxic atmospheres but not hypoxia conditions. In addition, the ROS level showed a mild increase when treated with FCP, FCP/HA, or FCP-Tph/HA, which might be caused by the Fenton reaction induced by the Fe^2+^ released from FCP in the acidic cytoplasm. These observations indicated that the photosensitizers, oxygen, and laser irradiation were indispensable for PDT. When treated with FCP-Tph/HA, the ROS concentration exhibited a high level in either normoxic or hypoxia atmosphere. Specifically, FCP-Tph/HA could produce a significantly higher level of ROS compared with Tph in a hypoxia atmosphere under laser irradiation (Figure 3F and Figure S7). This result indirectly revealed that FCP-Tph/HA could produce oxygen for enhanced PDT in tumor cells. The result of the reduced H_2_O_2_ level (tested with a Reactive Oxygen Species Assay Kit) of the cells being treated with an extra 10 mM H_2_O_2_ after being treated with FCP-Tph/HA also indicated that FCP-Tph/HA could decompose H_2_O_2_ into O_2_ for enhanced PDT in the cells (Figure S8).

Flow cytometry was used to detect cell apoptosis and necrosis at different conditions (Figure 3G, Figure S10, and Figure S11). As the results showed, after being treated with FCP, FCP/HA, and FCP-Tph/HA for 4 h without laser radiation, the cell viability was reduced by about 30% (if treated with FCP, FCP/HA, and FCP-Tph/HA without laser irradiation for 24 h, the cell viability was reduced by about 50%), which may be resulted from the cytotoxicity of FCP. Meanwhile, when treated with Tph without or with laser irradiation under a normoxic atmosphere, the cell viabilities were reduced by less than 5% or more than 60%, respectively. The results revealed that PDT presented a high effect for tumor treatment *in vitro* and laser irradiation was indispensable for PDT. However, in a hypoxia atmosphere, less than 20% of cell viability was reduced after being treated with Tph and laser irradiation. The result showed once again the importance of oxygen for a successful PDT 10,34. On the contrary, when treated by FCP-Tph/HA with laser radiation, ~70% of cell viability was reduced in both normoxic and hypoxia atmospheres, which was higher than the total cell viability decrement of treated by Tph under hypoxia atmosphere and FCP. The result indicated that FCP-Tph/HA presented a high efficacy antitumor activity by synergistic chemo-photodynamic therapy under the mimic anoxic tumor environment. In addition, the imaging results of the tumor cells treated with PBS, Tph with laser irradiation, FCP-Tph/HA, and FCP-Tph/HA with laser irradiation (all experiments were carried out under 1% O_2_ atmosphere) also indicated that FCP-Tph/HA showed high efficiency synergistic chemo-photodynamic therapy *in vitro* (Figure S12).

**Figure 4:**
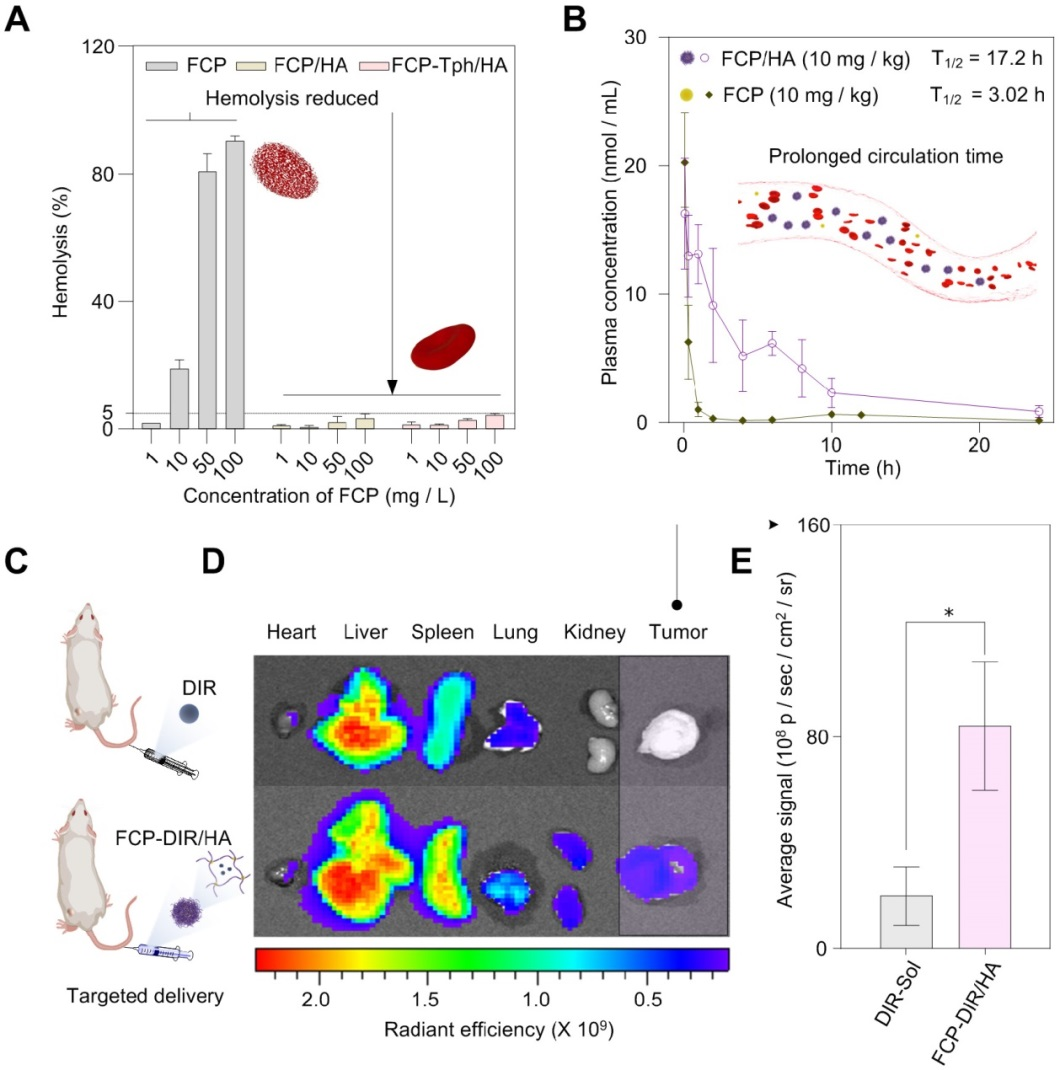
***In vivo* studies of the micelles.** A) The formation of nanomicelle weaken the hemolysis rate of FCP. B) FCP/HA extended the blood circulation time of FCP *in vivo* (n = 6). C) *In vivo* biodistribution of FCP-DIR/HA (n = 3). D) Fluorescent images at 24 h after drug injection; E) Quantitative results of relative organ and tumor accumulation at 24 h (n = 3). All values are presented as the mean ± SD. (*) p < 0.05 compared with DIR-Sol.

The hemolysis rate is an important index to evaluate the safety of drug candidates or nanoPDT 35. We then carried out the hemolysis test of FCP, FCP/HA, and FCP-Tph/HA. As shown in Figure 4A, and Figure S13, severe hemolysis occurred when the red blood cells were incubated with more than 10 mg/L FCP, suggesting side effects of FCP as a drug candidate. However, FCP/HA, and FCP-Tph/HA presented encouraging results. When the concentrations of FCP in FCP/HA and FCP-Tph/HA were higher than 50 mg/L, the hemolysis rate was still lower than 5%, indicating that HA could reduce the hemolysis rate of FCP.

Prolonged blood circulation time is one of the benefits of nanoPDT, which means a low dosing frequency 36. The pharmacokinetic profiles exhibited that FCP concentration in SD rats injected with FCP/HA declined much slower than ones injected with an equal amount of FCP (Figure 4B). The half-life (T_1/2_) of FCP by applying in FCP/HA was extended to 17.2 hours, compared with 3.0 hours by applying FCP (Table S4). Similarly, FCP/HA showed a lower total body clearance (CL) value, a higher area under the curve (AUC_0-24_), and a longer mean residence time (MRT_0-24_). The results revealed that the nanomicellar carrier prepared by the coordination-driven self-assembly could significantly prolong the circulation time of the cargo *in vivo* 37,38.

The biological distribution *in vivo* was studied in Balb/C mice bearing 4T1 tumor cells to assess the active targeting drug delivery ability of the nanomicelle. As shown in Figure 4C-E, the fluorescence intensity of tumors treated with DIR-labeled FCP/HA (referred to FCP-DIR/HA) was significantly higher than the tumors treated with DIR alone. This result suggested that HA contained nanoPDT was selectively taken up into tumor tissues, which was caused by the specific binding of HA and CD44 in the tumor tissue 27,31. These results indicated that the HA-containing nanoPDT could help transport drugs to the tumor site *in vivo*. In addition, the fluorescence intensity changes of tumors over time experiment indicated that the accumulation of the FCP-DIR/HA in the tumors reached the maximum after the injection of FCP-DIR/HA into mice for 24 h, which indicated that the best time for laser irradiation was 24 h after injection FCP-Tph/HA (Figure S14).

**Figure 5:**
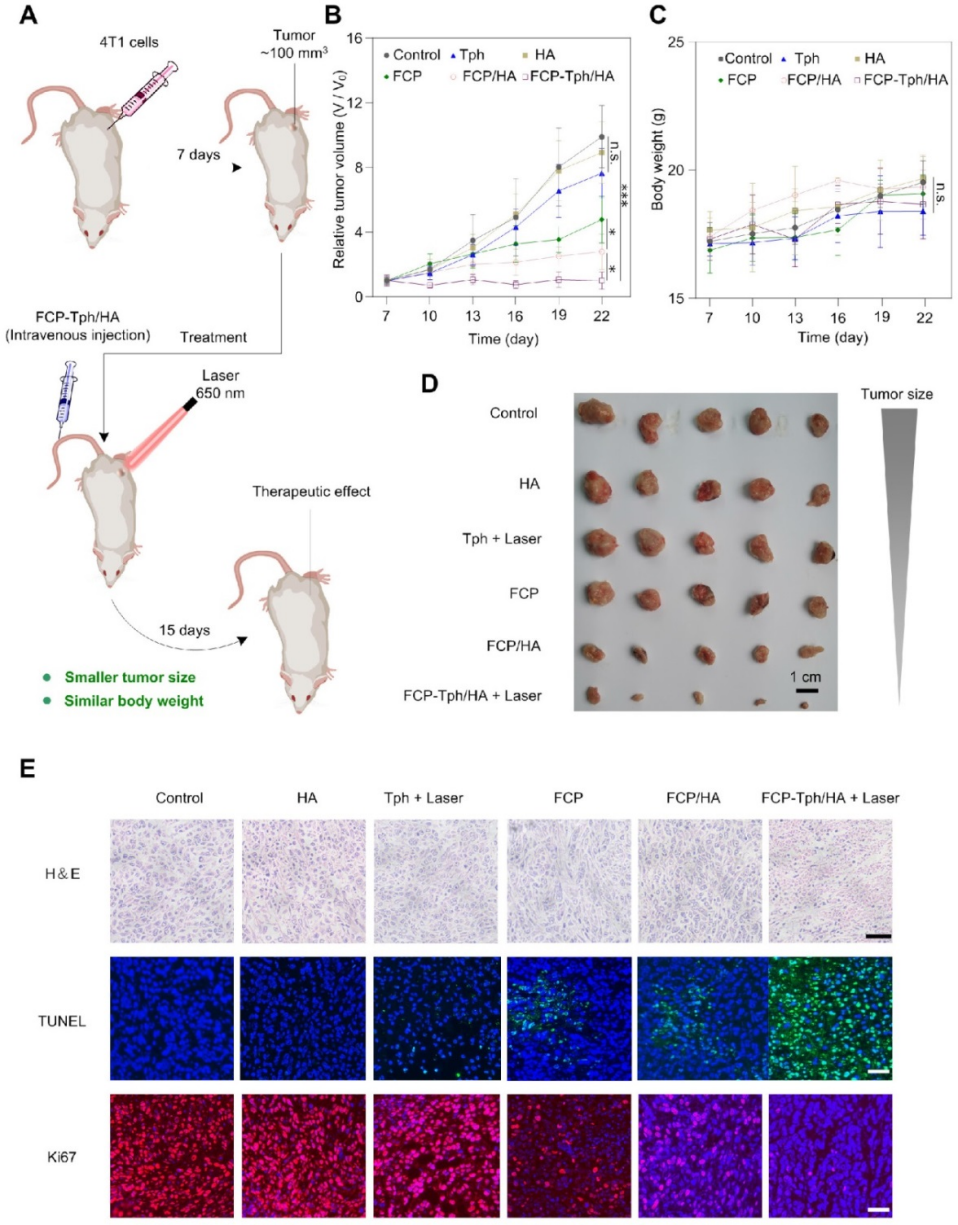
** FCP-Tph/HA improves the antiproliferative effects of FCP and Tph *in vivo*.** A). Schematic of tumor formation and injection of drugs to the tumor. B) Tumor growth profiles after treatment with different formulations. C) Plot of body weight versus time in tumor-bearing mice. (n = 5). (n.s.) *p* > 0.05, (*) *p* < 0.05, (***) *p* < 0.001 compared with normal saline (Control). D) Images of tumors after the final treatment. Scale bar, 1 cm. E) Histological analysis of tumor section stained with H&E, TUNEL, and Ki67 for mice with different treatment groups. Nuclei were stained with DAPI (blue), TUNEL (green), and Ki67 (red). Scale bars, 40 μm.

*In vivo* antitumor effects of FCP-Tph/HA were evaluated in the mice bearing 4T1 tumors (Figure 5A). The 4T1 tumor-bearing mice were divided into six groups randomly and treated with normal saline (Control), HA, Tph with laser 650 nm, FCP, FCP/HA, and FCP-Tph/HA with laser 650 nm every third day. As shown in Figure 5B, D, HA, and Tph alone treatment did not affect 4T1 tumor growth over 15 days. The results indicated that HA could only be used to the construction of a nano-carrier for drug delivery but not a therapeutic molecular. Besides, Tph with 650 nm laser irradiation showed high cytotoxicity to tumor cells *in vitro*. However, the untargeted aggregation and the hypoxic tumor microenvironment seriously hindered the *in vivo* PDT effect of Tph. In addition, the 4T1 tumors in the mice treated with FCP showed slightly slower growth than those as control, and the 4T1 tumors in the mice treated with FCP/HA were smaller than those treated with FCP. The result revealed again that FCP was a qualified potential chemotherapeutic agent for tumor treatment, and HA could tumor-targeted FCP delivery, thereby enhancing the antitumor efficiency of FCP *in vivo*. If treated with FCP-Tph/HA without laser irradiation, the tumors grew up to ~380 mm^3^ at day 22, which showed no significant difference in the sizes of the tumors between the tumors treated with FCP-Tph/HA (without 650 nm laser irradiation) and FCP/HA (Figure S15). However, FCP-Tph/HA with 650 nm laser irradiation displayed the strongest inhibition of 4T1 tumor growth in mice (about 100 mm^3^ at day 22), attributed to the synergistic chemo-photodynamic therapy of FCP-Tph/HA for solid tumor treatment* in vivo*. Moreover, little change in body weight was noticed in mice treated with normal saline, HA, Tph, FCP, FCP/HA, and FCP-Tph/HA (Figure 5C), indicating that all the systems administered did not show any major systemic toxicity. To further evaluate the antitumor efficiency under different conditions, hematoxylin and eosin (H&E) stain, terminal deoxynucleotidyl transferase dUTP nick end labeling (TUNEL), and Ki67 were used to stain the tumors under different treatment. The most severe morphological change and cell death from tumor slices were observed in the tumor treated with FCP-Tph/HA + Laser, indicating that FCP-Tph/HA + Laser showed the strongest antiproliferative effects over these 7 groups (Figure 5E and Figure S15C). No significantly histological changes were observed in hematoxylin-eosin (H&E)-stained tissue sections of the heart, liver, spleen, lung, and kidney, indicating the safety of these treatments (Figure S16).

## Conclusion

In summary, a nanomicellar PDT (FCP-Tph/HA) system was prepared for the synergistic chemo-photodynamic therapy of tumor treatment. The rational design of a multifunctional building block FCP enables a simple, rapid, and integrative self-assembly process to obtain the nanoPDT system. *In vitro* results revealed that FCP played a multifunctional role in inducing cancer cells apoptosis through a caspase-dependent pathway and generating oxygen by decomposing endogenous hydrogen peroxide for enhanced PDT in the mimetic hypoxic tumor microenvironment. Besides, FCP-Tph/HA exhibited an active tumor-targeting ability based on cellular uptake experiments and *in vivo* biodistribution experiments. In addition, the nanomicelle showed a reduced hemolysis rate and a prolonged blood circulation time supported by the experimental results of hemolytic tests and the pharmacokinetic experiments. Similarly, our *in vivo* results revealed that FCP-Tph/HA exhibited a superior antitumor activity, attributed to the synergistic chemo-photodynamic therapy. These results not only present a novel modular strategy for the combination of PDT with chemotherapy in tumor treatment but also contribute to a conceptual and practical paradigm for effective PDT using a rationally designed multifunctional ferrocene building block as an oxygen supplying agent.

## Supplementary Material

Supplementary figures and tables.
