# Supplementary material for: Self-assembly of nanomicelles with rationally designed multifunctional building blocks for synergistic chemo-photodynamic therapy
Source: Theranostics. 2022 Jan 31;12(5):2028–40. doi: 10.7150/thno.68563 (PMC8899564; doi:10.7150/thno.68563)
Supplement: Supplementary file 1 — Supplementary figures and tables. [file thnov12p2028s1.pdf]

.

Supporting Information for

Self-assembly of nanomicelles with rationally designed multifunctional building blocks for synergistic chemo-photodynamic therapy

Guidong Gong, Jiezhou Pan, Yunxiang He, Jiaojiao Shang, Xiaoling Wang, Yaoyao Zhang, Guolin Zhang, Fei Wang,* Gang Zhao,* and Junling Guo*

.

1. Materials

Acetylferrocene, (*R*)-(+)-amino-2-(methoxymethyl) pyrrolidine, $Pd(OAc)_2$, and Tph were purchased from Titan (Shang Hai, China). Coumarin 6, DIR, Hyaluronan (HA) (MW 10 kDa, 90 kDa, 80 – 150 kDa, and 180 kDa) were purchased from Dalian Meilun Biotech Co., Ltd (Dalian Chian), phosphate buffer saline (PBS) was bought from beyotime (Shanghai, China). Cell counting Kit-8 and Annexin V-FITC Apoptosis Detection Kit were purchased from Solarbio (Shanghai, China). Unless otherwise noted, materials were obtained from commercial suppliers and were used without further purification.

2. Instruments

Melting points were measured on a Meltemp melting point apparatus. Optical rotation was performed with the Perkin Elmer model 341 polarimeter. ^{1}H NMR spectra were recorded on Bruker AM400 NMR spectrometer, in which chemical shifts were signed in ppm from tetramethylsilane with the solvent resonance as the internal standard ($CDCl_3$, δ = 7.26 ppm). Spectra were reported as follows: chemical shift (δ ppm), multiplicity (s = singlet, d = doublet, t =triplet, q = quartet, m = multiplet), coupling constants (Hz), integration and assignment. ^{13}C NMR spectra were collected on commercial instruments (100 MHz) with complete proton decoupling. Chemical shifts are reported in ppm from the tetramethylsilane with the solvent resonance as internal standard ($CDCl_3$, δ = 77.0 ppm). MS spectra were recorded on a UPLC-Xevo™ TQMS system equipped with an ESI source. C, H, and N elemental determination were performed on a Euro EA 3000 elemental analyzer (Euro Vector, Italy). O_2 concentration was tested by JPSJ-605 Leizi Oxygen Dissolving Instrument (Titan, China). Size distribution and zeta potential were measured using Mastersizer 3000. TEM was measured with a TM-1000 Transmission Electron Microscope (Hitachi, Japan), Laser Scanning Confocal Microscopy was measured with Leica TCS SP8 Laser Scanning Confocal Microscopy (Germany). MW-RL-650 laser was supplied by Changchun Leishi Science and Technology Ltd. (China). *Ex vivo* biodistribution was measured with an IVIS spectrum small-animal

imaging system (IVIS Lumina Series III, PerkinElmer, USA). Blood concentration was measured with UPLC-Xevo™ TQ MS (Waters, USA).

3. FCP synthesis

Compound C1: acetylferrocene (10 mmol) and (*R*)-(+) - amino-2-(methoxymethyl) pyrrolidine were dissolved in dry benzene (100 mL) and were then filled in a flask equipped with a Dean-Stark apparatus. The red solution was refluxed over an oil bath for about 6 h and then carefully transferred into a Schlenk tube, with 5 Å molecular sieves (3.0 g) were introduced. The mixture was further refluxed for 6 h and then washed with n-hexane. Characterization data for:

C1: yield: 1.76 g (70%); m.p. 67.5-68.1 °C, $[\alpha]_D^{20}$ -430.8 (c 1.0 in $CHCl_3$); ^1H NMR (400 MHz, $CDCl_3$, 25 °C, TMS): δ = 4.67 [d, J = 1.3 Hz, 1H; H^2 (C_5H_4)], 4.59 [d, J = 1.4, 1H; H^5 (C_5H_4)], 4.35 – 4.23 [m, 2H; H^3, H^4 (C_5H_4)], 4.12 (s, 5H; C_5H_5), 3.50 (q, J = 7.2 Hz, 1H; CH) 3.38(s, 3H; OCH_3), 3.34-3.20 (m, 2H; OCH_2), 2.48 (dd, J = 17.1, 8.6 Hz, 1H; NCH_2), 2.19 (s, 3H;CH_3C=N), 2.04 (dt, J = 6.72 Hz, 1H; NCH_2), 1.94 - 1.80 (m, 2H; $CHCH_2CH_2$), 1.77 - 1.62 ppm (m, 2H; $CH_2CH_2CH_2$).

FCP: **C1** (297 mg, 1.0 mmol) was added to a methanolic (30 mL) solution containing $Pd(OAc)_2$ (224 mg, 1 mmol) and $NaOAc\cdot 3H_2O$ (140 mg, 1.0 mmol), and stirred at room temperature for 24 h. After the reaction completion, the resultant reaction mixture was dried under a high vacuum, and then the product was extracted into chloroform and passed through a SiO_2-column using PE/EA (4:1) as eluent. Finally, the purified **FCP** was obtained from the eluted solution via evaporating chloroform. Characterization data for:

FCP: yield: 0.27g (53%); m.p. 203.3-203.9 °C, $[\alpha]_D^{20}$ = -703.9 (**c** 1.0, $CHCl_3$); ^1H NMR (400 MHz, $CDCl_3$) δ = 4.83 [s, 1H; H^5 (C_5H_3)], 4.44-4.13 [m, 14H; H^5 + H^3 (C_5H_3) + C_5H_5], 3.62 (s, 1H; CH), 3.38 (s, 12H; CH_2OCH_3 + CH_2), 2.90 (s, 2H; CH_2), 2.26 (s, 6H; CCH_3), 2.15-1.60 ppm (m, 14H; CH_2); ^{13}C NMR (100 MHz, $CDCl_3$, 25°C, TMS): δ = 187.01 (C=N), 98.81 [C^1 (C_5H_3)], 85.47 [C^2 (C_5H_3)], 75.77 [C^5 (C_5H_3)], 73.76[C^3 (C_5H_3)], 70.62(C_5H_5),

67.40 [C^4 (C_5H_3)], 65.61 (OCH_2), 59.06 (NCH), 56.62 (OCH_2), 54.82(NCH_2), 26.66 (CH_2CH_2), 22.25 (CH_2CH_2), 15.20 ppm(C=NCH_3); ^{13}C NMR (101 MHz, $CDCl_3$) δ 186.6 (C=N), 178.2 (CH_3C), 85.5 [C^1 (C_5H_3)], 75.7 [C^2 (C_5H_3)], 75.0 [C^5 (C_5H_3)], 70.6 [C^3 (C_5H_3)], 67.7 (C_5H_5), 65.5 (OCH_2), 62.8 (NCH), 59.0 (OCH_2), 54.4 (NCH_2), 26.9 (CH_2CH_2), 24.3 (CCH_3), 22.2 (CH_2CH_2), 15.0 ppm (C=NCH_3); MS (ES+): calcd for $C_{40}H_{52}Fe_2N_4O_6Pd_2$ [M + Na]$^+$: 1031.0548, Found: 1031.0587. Anal. calcd for $C_{40}H_{52}Fe_2N_4O_6Pd_2$: C, 47.59; H, 5.19; N, 5.55. Found: C, 47.58; H, 5.14; N, 5.58.

4. Scheme of synthesis, supplementary tables, and figures.

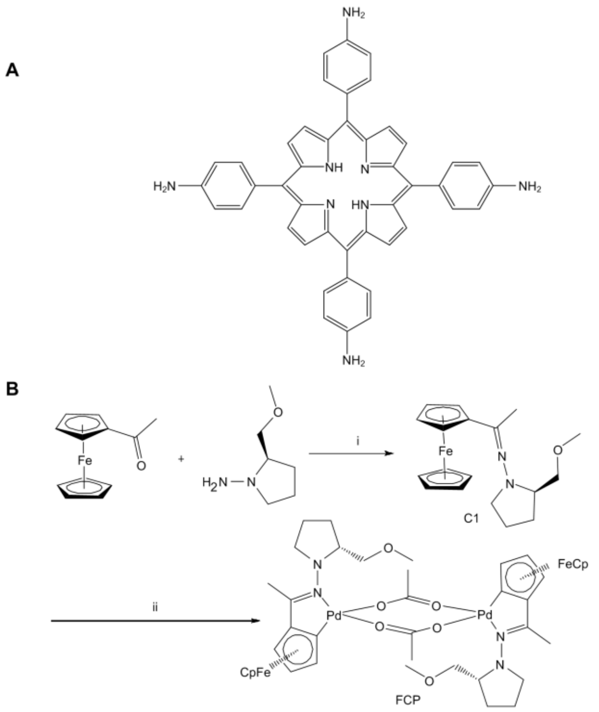

Figure S1. A) Structural formula of Tph. B) Overview of synthesized compounds FCP. i) $C_6H_5CH_3$, 110°C, 24 h. ii) $Pd(OAc)_2$, NaOAc, MeOH, r.t., 24 h.

.

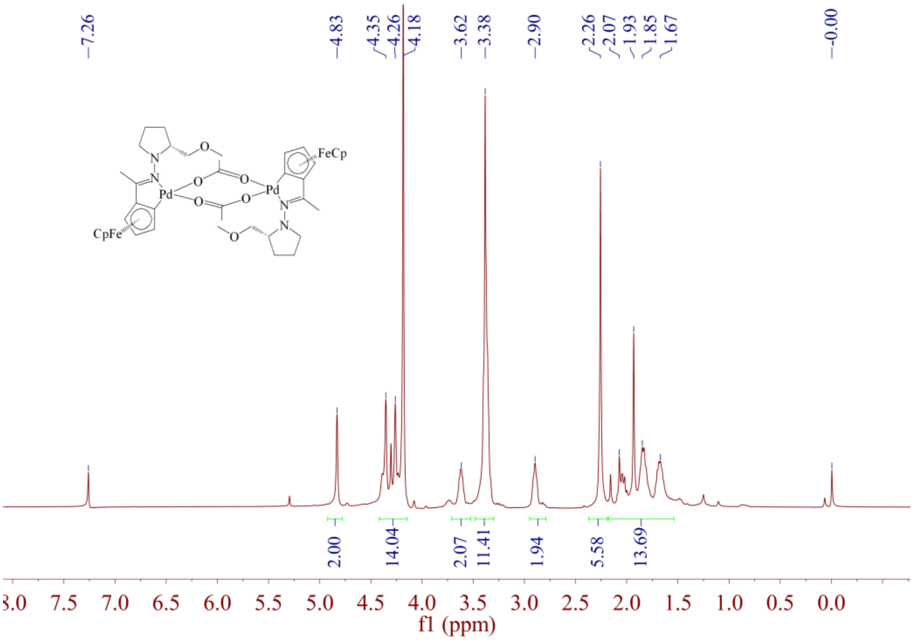

Figure S2. ^{1}H NMR Spectrum of F**CP** ($CDCl_3$, 400 MHz).

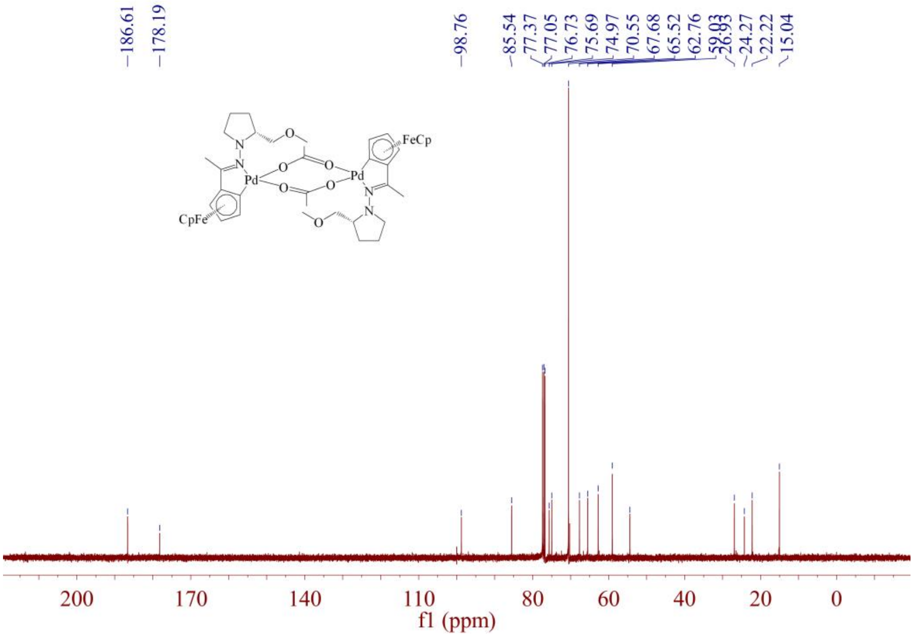

Figure S3. ^{1}H NMR Spectrum of F**CP** ($CDCl_3$, 400 MHz).

Table S1. The average size of the nanomicelles.

**Number**	**Relative molecule weight of HA (kD)**	**Feed mass ratio (HA:FCP)**	**Size (nm)[a]**
**1**	10	2.9:1	172.7
**2**	10	5.9:1	197.9
**3**	10	11.8:1	191.7
**4**	90	2.9:1	282.1
**5**	90	5.9:1	577.8
**6**	90	11.8:1	627.0
**7**	800 ~ 1 500	2.9:1	950.7
**8**	800 ~ 1 500	5.9:1	1918.3
**9**	800 ~ 1 500	11.8:1	4375.7
**10**	1 800	2.9:1	2990.0
**11**	1 800	5.9:1	5825.3
**12**	1 800	11.8:1	1083.3

[a] data was obtained by DLS.

Table S2. The size, zeta potential, ER, and DL of nano micelles prepared with HA (10 KDa).

**Number**	**HA:CP**		**Size (nm)[a]**	**PDI[a]**	**Zeta (mV)[a]**	**ER(%)[b]**	**DL(%)[b]**
	**Mole ratio (carboxyl in HA : CP)**	**Feed mass ratio**					
**1**	7:1	2.9:1	172.7	0.096	-28.4	13.78	46.33
**2**	14:1	5.9:1	197.7	0.053	-34.7	12.82	86.78
**3**	28:1	11.8:1	191.7	0.142	-30.2	7.13	90.63

[a] data are collected by Malvin potentiometer.

[b] data are collected by ICP-OES.

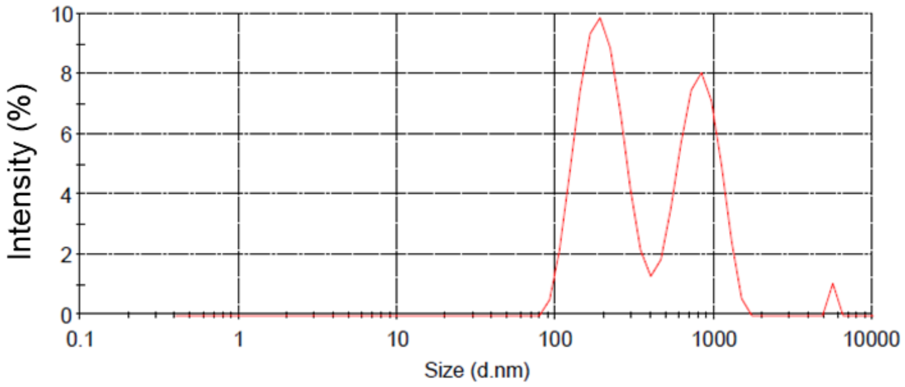

Figure S4. Size distribution of FCP/HA after being stimulated by HAase for 24 hours at 37 ^{o}C.

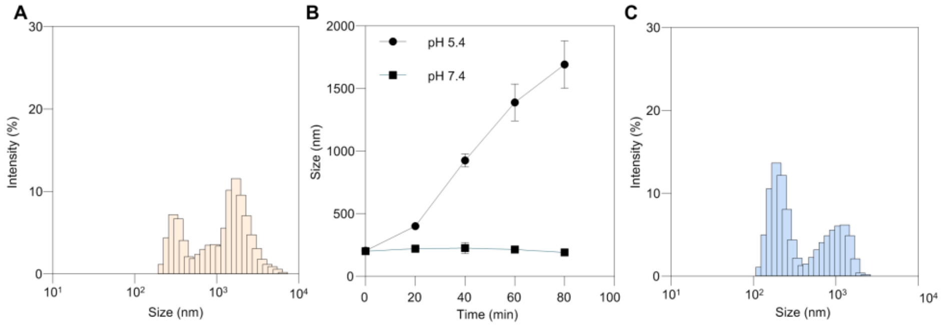

Figure S5. DLS analysis of FCP-Tph/HA. A) Size distribution of FCP-Tph/HA treated with acid (pH = 5.4) for 12 h. B) Size change of FCP-Tph/HA at different incubation time in pH = 5.4 and pH = 7.4. C) Size distribution of FCP-Tph/HA treated with GSH (5 mM) for 12 h.

.

Table S3. IC_{50} (μg/mL) for FCP, FCP/HA, HA+CP/HA, and cisplatin against NIH 3T3, MDA-MB-231, and 4T1 cell lines.

**Compounds**	**IC_{50} (μM)**		
	**NIH 3T3**	**MDA-MB-231**	**4T1**
**CP**	5.41 ± 1.0	1.00 ± 0.06	2.69 ± 0.07
**FCP/HA**	8.92 ± 0.23	3.31 ± 0.25	5.04 ± 0.44
**HA+FCP/HA**	8.43 ± 0.73	10.4 ± 0.46	9.20 ± 0.14
**Cisplatin**	41.5 ± 3.3	10.9 ± 0.92	11.4 ± 0.57

.

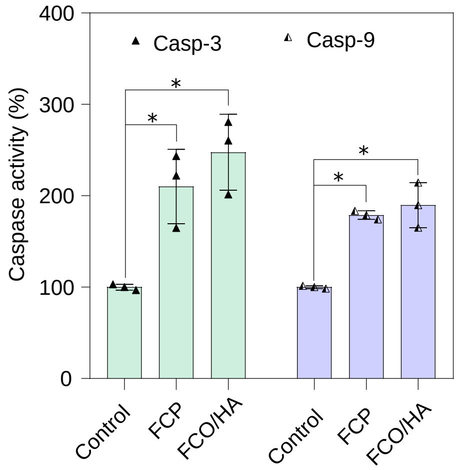

Figure S6. Caspase 3 and Caspase 9 activation in 4T1 cells after treatment with FCP or FCP/HA for 12 h.

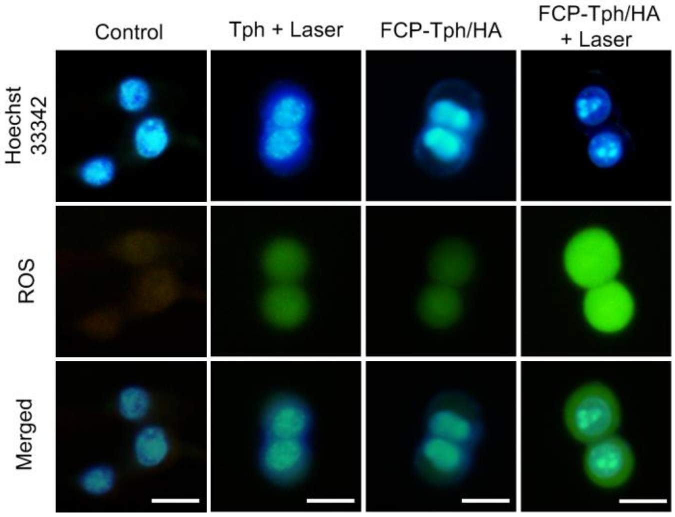

Figure S7. Representative images of intracellular ROS level after being treated with PBS (Control), Tph with laser irradiation, FCP-Tph/HA, and FCP-Tph/HA with laser irradiation. All the measured cells used in this experiment were treated under hypoxic conditions. Scale bar, 10 μm.

.

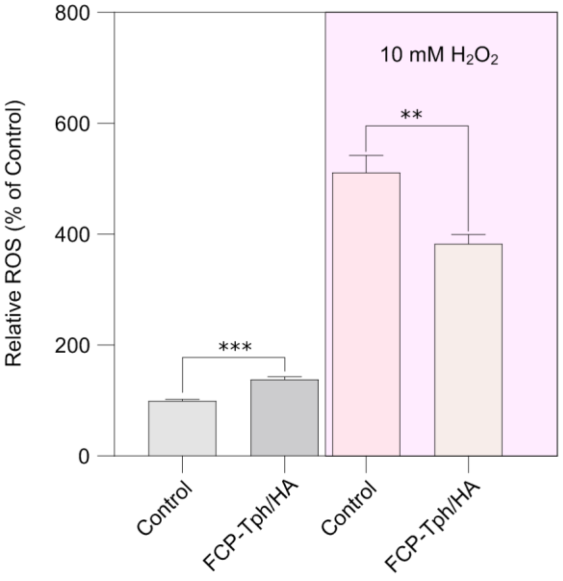

Figure S8. The relative ROS level variation of cells treated with an extra 10 mM H_2O_2 after being treated with or without FCP-Tph/HA ([FCP] = 10 μM).

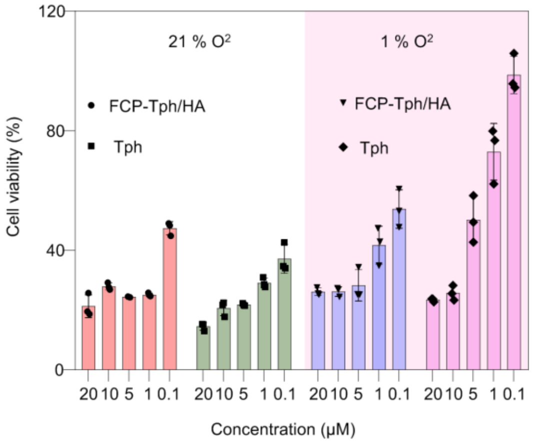

Figure S9. Cell viability of MDA-MB-231 cells treated with Tph or FCP-Tph/HA at different concentrations (the concentration of FCP-Tph/HA means the molar concentration of FCP and Tph in FCP-Tph/HA) for 4 hours under normoxic (21%) or hypoxic (1% O_2) conditions upon 650 nm light irradiation (200 mW/cm^2) for 10 min.

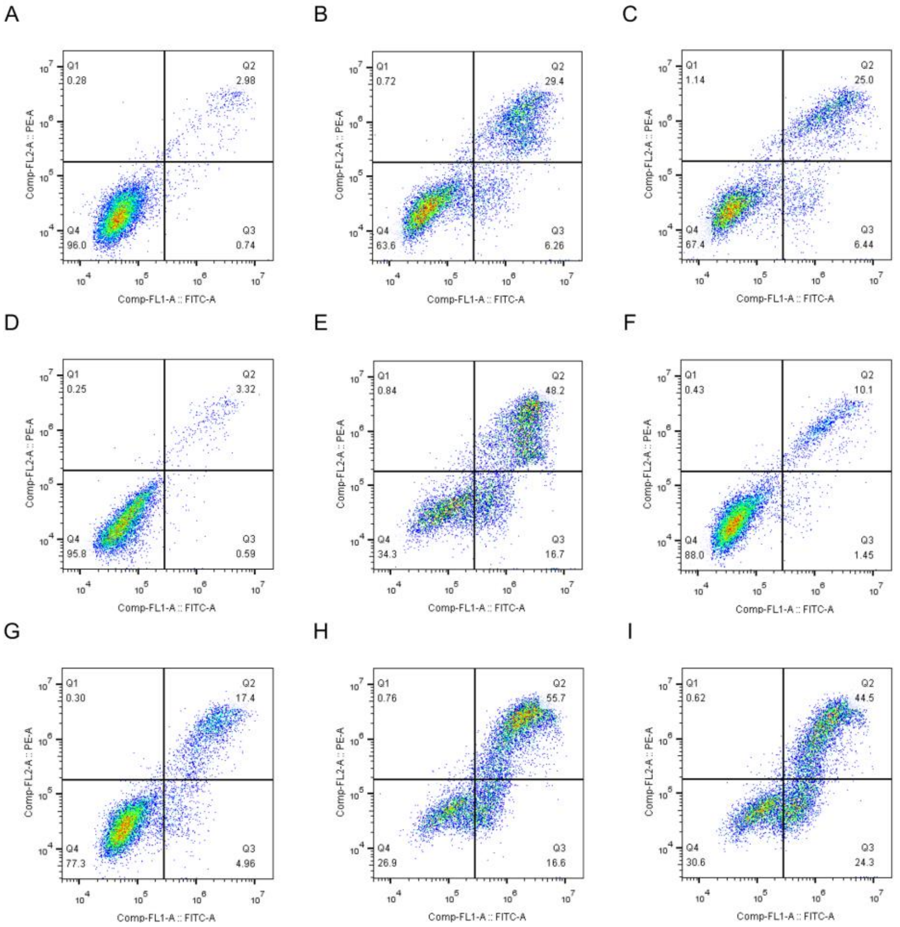

Figure S10. Apoptosis assay by flow cytometry; A) Control, B). FCP; C). FCP/HA; D). Tph; E). Tph + PDT; F). Tph + PDT - O_2 G). FCP-Tph/HA; H). FCP-Tph/HA + PDT; I) FCP-Tph/HA + PDT - O_2.

.

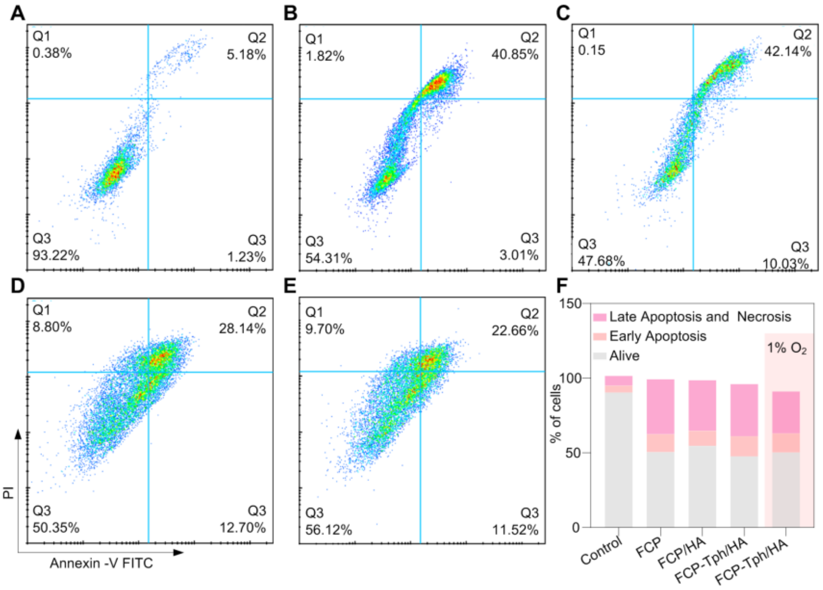

Figure S11. Apoptosis assay by flow cytometry of cells under different treatments. A) Control. B) FCP. C) FCP/HA. D) FCP-Tph/HA. E) FCP-Tph/HA – O_2. F) Different percent obtained from the apoptotic study.

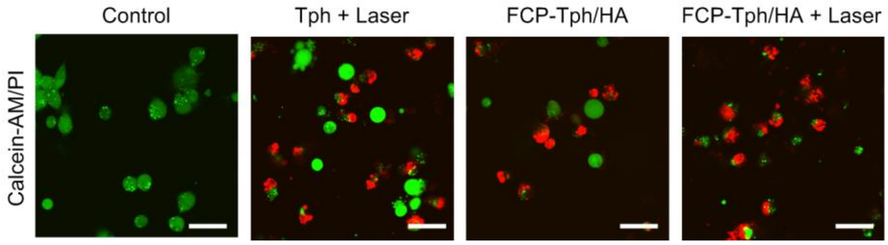

Figure S12. Representative images of 4T1 cells under hypoxia atmosphere (1% O_2) and stained with Calcein-AM (green, live cells) and PI (red, dead cells). Scale bare, 20 μm.

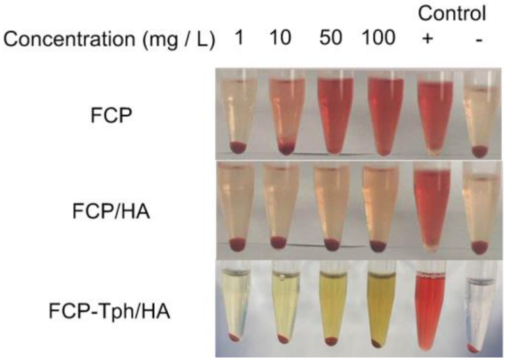

Figure S13. The hemolysis of RBCs after incubated with different concentrations of FCP, FCP/HA, and FCP-Tph/HA (the concentrations in the figure were indicated the concentration of FCP) for 4 hours at 37°C.

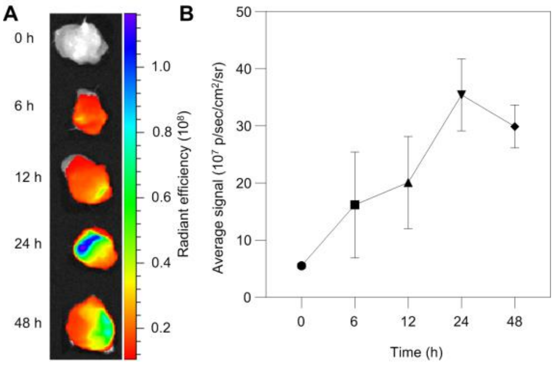

Figure S14. The accumulation of FCP-DIR/HA in the tumors over time.

Table S4. Pharmacokinetic results of FCP and FCP/HA. (n=6)

Formulations	Determined	$T_{1/2}$ / h	CL / mL/L/h	AUC_{0-24} / nmol/mL/h	MRT_{0-24} / h
**FCP**	FCP	3.02 ± 1.7	0.85 ± 0.19	11.03 ± 1.9	2.20 ± 1.0
**FCP/HA**	FCP	17.19 ± 5.3	0.18 ± 0.14	73.38 ± 28	4.43 ± 2.3

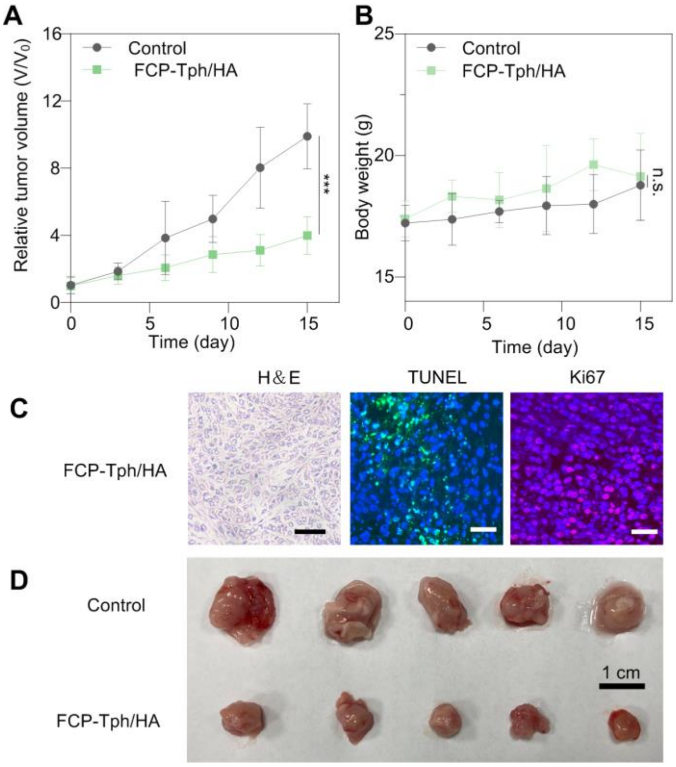

Figure S15. The antiproliferative effect of FCP-Tph/HA without 650 nm laser irradiation *in vivo*. A) Tumor growth profiles after treatment with FCP-Tph/HA. B) Plot of body weight versus time in tumor-bearing mice (n = 5). (n.s.) $p > 0.05$, (***) $p < 0.001$ compared with Control. C) Histological analysis of tumor section stained with H&E, TUNEL, and Ki67 for mice with FCP-Tph/HA treatment group. Nuclei were stained with DAPI (blue), TUNEL (green), and Ki67 (red). Scale bar, 40 μm. D) Image of tumors after the final treatment. Scale bar, 1 cm.

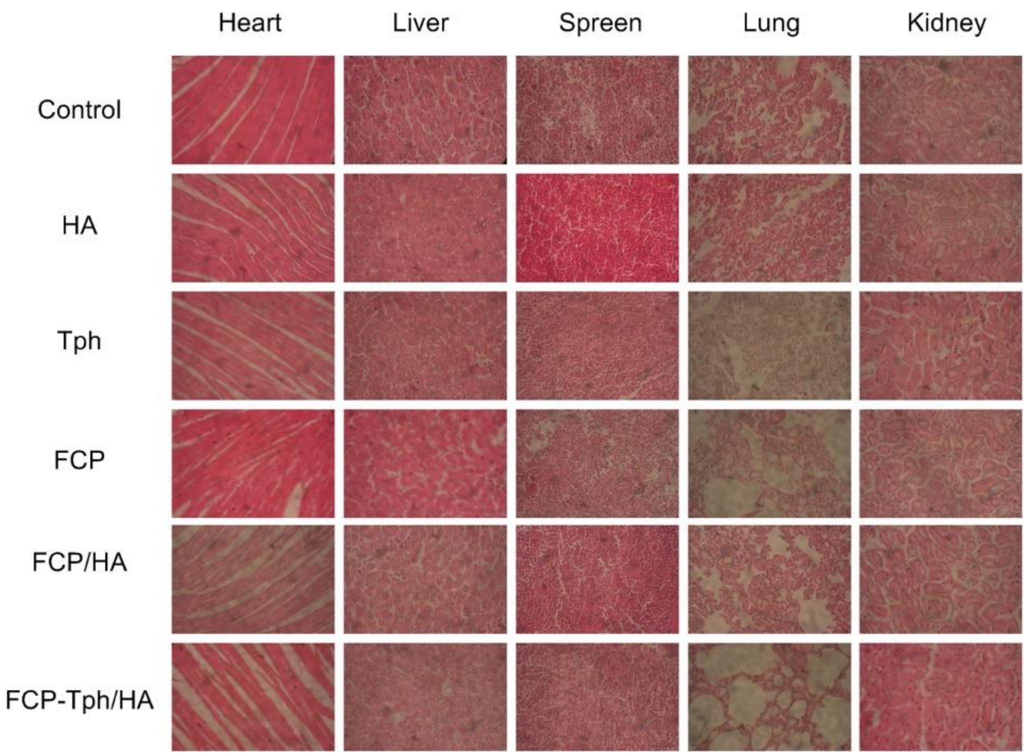

Figure S16. H&E staining images of heart, liver, spleen, lung, and kidney after the treatments (Nikon Ti-E microscope (× 400)).
